# Meaningful Communication but not Superficial Anthropomorphism Facilitates Human-Automation Trust Calibration: The Human-Automation Trust Expectation Model (HATEM)

**DOI:** 10.1177/00187208231218156

**Published:** 2023-12-02

**Authors:** Owen B. J. Carter, Shayne Loft, Troy A. W. Visser

**Affiliations:** 1 2720 The University of Western Australia, Australia

**Keywords:** human-automation teaming, trust, team communication, anthropomorphism, naturalistic decision making

## Abstract

**Objective:**

The objective was to demonstrate anthropomorphism needs to communicate contextually useful information to increase user confidence and accurately calibrate human trust in automation.

**Background:**

Anthropomorphism is believed to improve human-automation trust but supporting evidence remains equivocal. We test the Human-Automation Trust Expectation Model (HATEM) that predicts improvements to trust calibration and confidence in accepted advice arising from anthropomorphism will be weak unless it aids naturalistic communication of contextually useful information to facilitate prediction of automation failures.

**Method:**

Ninety-eight undergraduates used a submarine periscope simulator to classify ships, aided by the Ship Automated Modelling (SAM) system that was 50% reliable. A between-subjects 2 × 3 design compared SAM *appearance* (anthropomorphic avatar vs. camera eye) and voice *inflection* (monotone vs. meaningless vs. meaningful), with the *meaningful* inflections communicating contextually useful information about automated advice regarding certainty and uncertainty.

**Results:**

*Avatar* SAM appearance was rated as more anthropomorphic than camera *eye*, and *meaningless* and *meaningful* inflections were both rated more anthropomorphic than *monotone*. However, for subjective trust, trust calibration, and confidence in accepting SAM advice, there was no evidence of anthropomorphic appearance having any impact, while there was decisive evidence that *meaningful* inflections yielded better outcomes on these trust measures than *monotone* and *meaningless* inflections.

**Conclusion:**

Anthropomorphism had negligible impact on human-automation trust unless its execution enhanced communication of relevant information that allowed participants to better calibrate expectations of automation performance.

**Application:**

Designers using anthropomorphism to calibrate trust need to consider what contextually useful information will be communicated via anthropomorphic features.

## Introduction

The incorporation of human-like features into automation is known as anthropomorphism, the term being a conjunction of the Greek words for ‘human’ (ánthrōpos, ἄνθρωπος) and ‘form’ (morphē, μορφή). There are mixed opinions within the scientific literature about the potential of anthropomorphism to improve human-automation team (HAT) trust. Several literature reviews (de Visser et al., 2019; Hoff & Bashir, 2015; Schaefer et al., 2016) describe multiple factors contributing towards HAT trust, including human operator characteristics (e.g., expertise, personality, age, education, and culture), and contextual factors (e.g., task complexity, workload, competing demands, and decision risk), but automation characteristics are generally considered most influential (e.g., reliability, performance, transparency, understandability, predictability, and anthropomorphism).

Some researchers describe anthropomorphism as ‘the critical variable’ for HAT trust formation and maintenance (e.g., de Visser et al. 2016, p. 331). Fink (2012) suggested that—at a fundamental level—humans are best-suited to interact with other humans and therefore have a natural preference to interact with automation in a similar way. Others argue that if humans recognise social cues resulting from anthropomorphism, they instinctively apply social heuristics (Christoforakos et al., 2021), potentially resulting in expectations that the automation possesses humanlike capabilities and is hence more trustworthy (Nass & Moon, 2000). Epley et al. (2007) suggest humans tend to ascribe human qualities to machines as a heuristic to make them explainable and predictable.

On this basis, many researchers have added anthropomorphism to automation to elicit social reactions in human teammates as a means of bolstering HAT trust outcomes (Bass et al., 2013; Hancock et al., 2011; Pak et al., 2014; Yee et al., 2007). This has been attempted by manipulating both appearance (e.g., incorporating human faces) and communicating sociability (e.g., incorporating emotional responses, encouragement dialogues and apologies for errors). However, empirical evidence supporting the impact of anthropomorphism remains mixed. Yee et al. (2007) conducted a meta-analysis of 25 studies published from 1996–2005 on the impact of automation featuring humanlike faces. They concluded that, compared to absence of anthropomorphism, presence of anthropomorphism produced a small positive effect on subjective trust (e.g., self-reported likeability and trust) (*R*^2^ = .02) and behavioural trust measures (e.g., task performance) (*R*^2^ = .02). They also reported a smaller, positive effect on subjective trust measures when comparing low versus high anthropomorphic realism (*R*^2^ = .01) but no corresponding difference for behavioural trust. Overall, Yee et al. concluded that while humans appreciate the aesthetic of anthropomorphism, level of realism matters little, and the authors expressed some concern that the small effects associated with anthropomorphism may be largely explained by the *Hawthorne Effect*—where participants in experimental settings behave in a socially desirable manner consistent with what they perceive the experimenters are seeking (see Adair, 1984).

Since publication of this metaanalysis, a corpus of additional studies has been conducted. Some studies have reported a positive effect of anthropomorphism on subjective and behavioural measures of trust. For instance, Pak et al. (2012) reported that a phone-based medical app featuring a photograph of a young, female doctor was more trusted (self-reported trust, automation reliance) by young participants—but not older participants—compared to the same app with no human photograph. Similarly, Waytz et al. (2014) added nonvisual anthropomorphic features to an automated vehicle by assigning a name, sex, and human voice and found it increased self-reported trust of the vehicle, and objective confidence in the automation as indexed by increased startle reflex and heart rate during an unavoidable vehicle collision.

However, consistent with the conclusions of Yee et al. (2007), many studies comparing presence to absence of anthropomorphism failed to find an effect. For instance, no effect on behavioural trust (e.g., attention allocation and automation reliance) was observed for participants operating a simulated bomb disposal robot (Gray et al., 2022), an aerial drone searching for potholes (Okamura & Yamada, 2020), or an industrial robot (Onnasch & Hildebrandt, 2022). Some studies even reported that anthropomorphism reduced self-reported trust when using a computer-based pedagogical tutor (Sträfling et al., 2010) or when operating an industrial robot whose face participants found ‘distracting’ (Roesler et al., 2020).

Comparisons of low versus high anthropomorphic realism have also yielded mixed results. One study comparing six levels of facial realism reported that participants playing an investment game subjectively preferred automation with more realistic faces, but this made no difference behaviourally to how much money participants entrusted to robotic teammates (Mathur & Reichling, 2016). Others reported that compared to absence of anthropomorphism, automation depicted with a human face or avatar was subjectively trusted more for a collaborative image classification task (Jensen, 2021) and a collaborative HAT computer game (Kulms & Kopp, 2019). However, these studies found no behavioural differences between avatar and human depictions (e.g., automation reliance), suggesting level of anthropomorphic realism had no appreciable effect.

In summary, the bulk of evidence suggests anthropomorphism has some kind of effect on trust, but this effect is inconsistent, often small, and usually confined to subjective, rather than behavioural, trust measures. Unidentified differences across task domains and experimental designs could have contributed to the mixed findings. Nonetheless, the lack of supporting evidence brings into question the theoretical mechanism by which anthropomorphism is thought to modulate human trust in automation. As discussed, some theorise that if humans recognise social cues resulting from anthropomorphism, they tend to apply social heuristics (Christoforakos et al., 2021; Fink, 2012), resulting in assumptions that the automation is possessed of humanlike capabilities and hence is more trustworthy (Nass & Moon, 2000). We argue that in the absence of relevant information, anthropomorphic automation may lead to higher subjective trust, but it is unlikely to improve behavioural trust. We further argue that *trust calibration* (i.e., the accuracy of automation use) is the most relevant behavioural trust outcome because it directly reflects automation use and disuse rates (Lee & See, 2004; Parasuraman & Riley, 1997).

**Figure 1.**
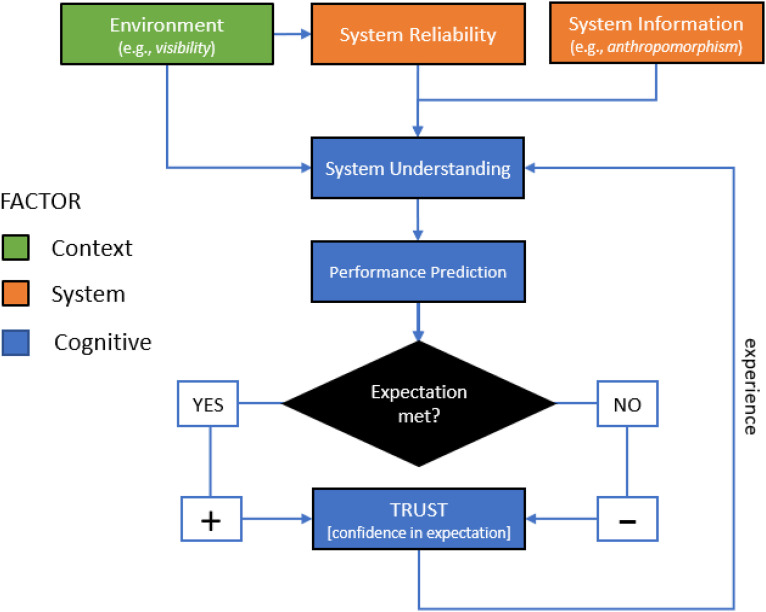
The Human-Automation Trust Expectation Model (HATEM) proposes that human trust in automation becomes increasingly calibrated over time through human *understanding* of automation *reliability*, allowing the human to dynamically *predict* automation *performance* within environmental context. Trust is thus defined by HATEM as the difference (closeness) between experienced automation reliability and expected automation reliability (prediction error). Observed automation performance is evaluated against *expectation* and either increases or decreases *confidence* in future predictions (trust), thereby refining *understanding*. Repeated experience allows testing and modification of expectations that can improve trust calibration.

The customary definition of HAT trust is the extent to which a human is willing to become vulnerable in the figurative hands of automation (Lee & See, 2004). The present study uses a complementary but more specific definition that operationalizes HAT trust as a human’s confidence in their ability to dynamically predict automation performance errors (i.e., to not be surprised). This is derived from the Human-Automation Trust Expectation Model (HATEM) proposed by Carter (2023), based upon cognitive science assertions that the human brain is essentially a ‘prediction machine’ (Clark, 2013). HATEM conjectures that if human expectations of automation performance are disappointed, confidence in future expectations will decrease, thereby diminishing HAT trust. If expectations are met, confidence will increase, and HAT trust will be reinforced. Thus, HATEM describes competent HAT trust calibration as expertise in automation error prediction, based upon nuanced understanding of how automation performs under various conditions. HATEM complements previous models that suggest HAT trust is multifactorial, and affected by a wide range of operator characteristics, contextual factors, and automation characteristics (e.g., Hoff & Bashir, 2015). Specifically, HATEM asserts these other known factors affect HAT trust solely through their ability to facilitate more accurate human predictions of automation performance errors. For instance, reliability is a major predictor of HAT trust (Hancock et al., 2011; Lee & See, 2004; Schaefer et al., 2016). HATEM posits that the contribution of reliability to HAT trust—in isolation—is based purely upon the information it confers about probability of automation failure, rather than any nuanced understanding that helps to predict the contexts under which such failures might occur. Consequently, HATEM predicts that in the absence of nuanced understanding of automation performance under various conditions, high reliability will produce more brittle HAT trust when errors are unanticipated compared to when they are predicted (Figure 1).

In the case of anthropomorphism, HATEM makes two very specific predictions: anthropomorphism will only improve trust calibration if (1) its execution improves communication of contextually useful information and (2) such information facilitates human prediction of automation failures. A corollary of these predictions is that superficial anthropomorphism of automation—such as manipulation of appearance—may result in naïve humans applying social heuristics of humanlike capability to automation, resulting in initially greater performance expectations and higher subjective trust ratings. However, HATEM predicts this effect will quickly dissipate under the inexorable onslaught of expectations disappointed, modified, retested, and honed by experience. In line with this, van Pinxteren et al. (2019) observed that novice participants self-reported greater trust for robots with humanlike head movements compared to those without head movements, but this effect was negated once greater understanding was developed of the robot capabilities.

HATEM also predicts work systems that communicate automation error probability—via any medium (e.g., anthropomorphic or not)—will yield better user confidence in expectations and calibrated trust. A true advantage of anthropomorphism may therefore be its ability to communicate to humans efficiently through an intuitive and naturalistic medium. For instance, Beller et al. (2013) investigated the use of an anthropomorphic symbol to communicate semi-automated vehicle uncertainty, represented by a shrugging cartoon character. With anthropomorphism present, participants demonstrated greater subjective trust, enhanced trust calibration behaviour, and quicker responses compared to when anthropomorphism was absent. HATEM suggests that rather than the anthropomorphic symbol improving trust due to its humanlike appearance, the trust stemmed from its ability to naturalistically communicate useful information to assist the human teammate to predict automation error more accurately.

### Current Study

HATEM makes clear and testable predictions about how anthropomorphism can engender better-calibrated HAT trust, and moreover, when it is likely to fail. The present study evaluated these predictions. An online submarine periscope simulator was used that tasked participants to categorise ships by class and heading. Task uncertainty, a prerequisite when studying trust (see Kohn et al., 2021), was manipulated by introducing six levels of ship visibility. Participants were advised by the Ship Automated Modeller (SAM) when categorising each ship. Anthropomorphism was introduced by assigning the automation a human name (SAM), sex (male), and human voice, emulating the study of Waytz et al. (2014). As described below, two further manipulations of anthropomorphism were introduced to SAM using a 2 × 3 between-subjects design.

#### Anthropomorphic Appearance

Anthropomorphism studies commonly manipulate automation appearance and report greater subjective trust of anthropomorphic compared to nonanthropomorphic manipulations of automation. Several studies also report avatars engender equivalent, or even superior, levels of subjective trust to photographs of humans (de Visser et al., 2017; Jensen, 2021; Kulms & Kopp, 2019; Sträfling et al., 2010). Others stress the importance of humanoid automation retaining some ‘robotness’ (Culley & Madhavan, 2013; DiSalvo et al., 2002). As such, the high anthropomorphism condition in the present study used a realistic image of a humanoid robot (*avatar*). The low anthropomorphism condition used an image of a disembodied robot camera eye with negligible anthropomorphic features (*eye*). The findings of Yee et al. (2007) suggest the greater anthropomorphism of *avatar* could engender assumptions of greater human-like competence resulting in increased subjective trust ratings compared to *eye*. Crucially, HATEM predicts that while participants may *perceive* greater subjective trustworthiness in SAM *avatar* than *eye*, anthropomorphic appearance will have no discernible impact on behavioural trust as measured by trust calibration (i.e., accuracy of automation use) or the degree of confidence when accepting SAM advice because appearance provides no information to help predict automation reliability.

#### Anthropomorphic Voice

Several studies report manipulating anthropomorphism by comparing automation with a human voice versus no voice (Christoforakos et al., 2021; e.g., Okamura & Yamada, 2020; Waytz et al., 2014). However, we are unaware of previous studies that have systematically manipulated the anthropomorphism of the voice itself. Here, we manipulated the inflection of SAM’s voice to create three conditions, but critically only two of them varied in level of anthropomorphism. In the nonanthropomorphic voice condition, SAM communicated with a monotone voice devoid of inflection (*monotone*). In the first anthropomorphic voice condition, SAM communicated with the same voice but included declarative downward inflections and interrogative upward inflections that occurred equally at random (*meaningless*). In the second anthropomorphic voice condition, SAM communicated with the same declarative downward or interrogative upwards inflections, but these naturalistically communicated automation certainty (declarative downward inflection) and uncertainty (interrogative upward inflection), respectively—and hence were meaningful in the sense that they contextually communicated likely automation reliability (*meaningful*).

The findings of Yee et al. (2007) suggest that *meaningless* and *meaningful* SAM will be subjectively trusted more than *monotone* SAM because they are more anthropomorphic in their resemblance to human-like inflections. Similarly, *meaningless* SAM and *meaningful* SAM should be equally trusted because they are inflected in the same anthropomorphic style. However, HATEM predicts only *meaningful* SAM will improve behavioural trust as measured by trust calibration (i.e., accuracy of automation use) and confidence when accepting SAM advice, because neither *monotone* nor *meaningless* SAM communicate useful information to assist participants to predict automation errors, whereas *meaningful* SAM inherently does.

HATEM further predicts that the behavioural trust engendered by *meaningful* SAM will improve over task time as individuals refine their understanding of the degree to which observed automation reliability matches their expectations (i.e., refinement of prediction error). Thus, HATEM predicts that trust calibration (accuracy of automation use), and confidence when accepting SAM advice, will both increase over time for individuals provided *meaningful* SAM. In contrast, trust calibration and confidence in *monotone* or *meaningless* SAM will not change over time because neither of these conditions allow individuals to refine their prediction error.

A positive correlation between trust calibration (accuracy of automation use) and confidence when accepting SAM advice would be indicative of convergent validity for these behavioural trust measures. HATEM predicts that subjective trust, trust calibration (behavioural trust), and confidence in automated advice should all be positively correlated because they are driven by the difference (closeness) between experienced automation reliability and expected automation reliability (prediction error).

## Method

### Participants

Ninety-eight undergraduate students at The University of Western Australia participated in the experiment in exchange for course credit. They had a mean age of 20.7 years (*SD =* 6.1), 68% were female, 31% male, and 1% other. The research was approved by the Human Research Ethics Office at the University of Western Australia. Informed consent was obtained from each participant.

### Material

**Figure 2.**
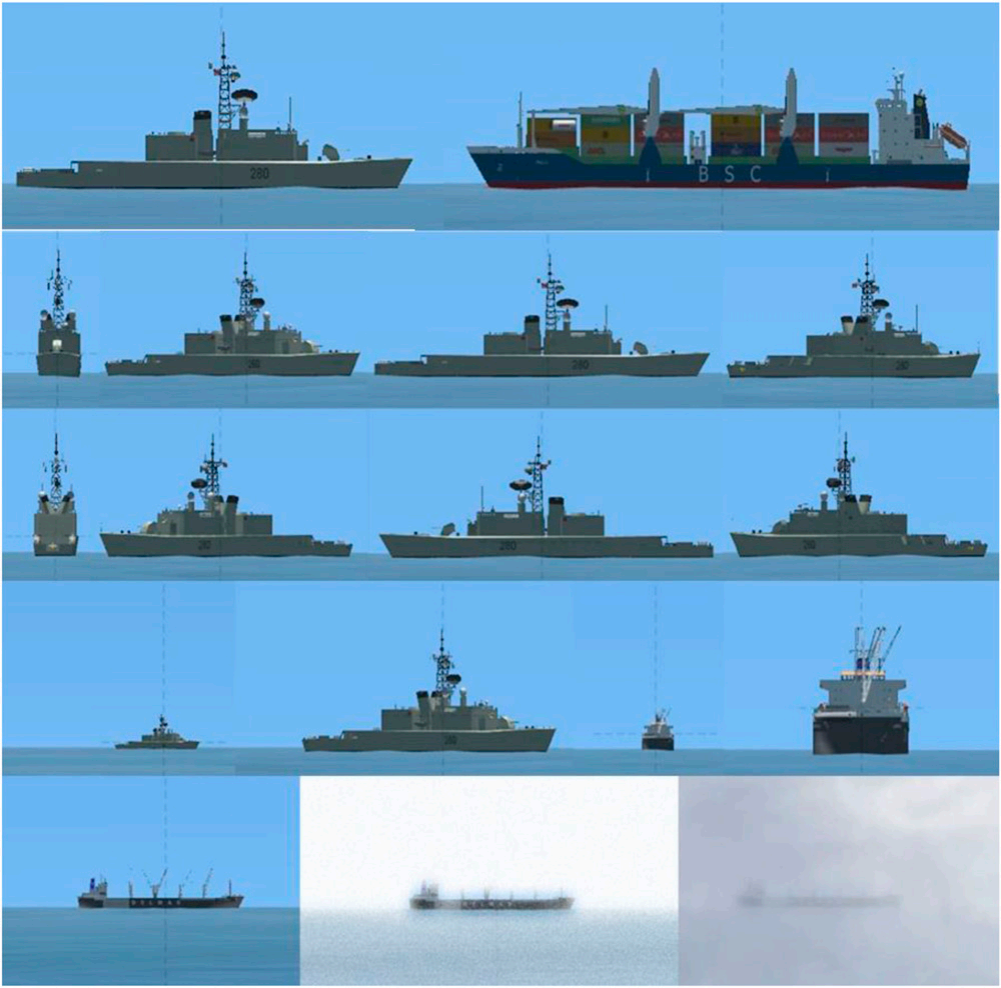
Ninety-six periscope images varied by all possible combinations of two ship classes (top row), eight headings (second and third rows), two ranges (fourth row), and three visibilities (bottom row).

The submarine periscope simulator was developed using PsyToolkit (Stoet, 2010, 2016). It presented participants with 96 ship images consisting of every combination of two ship classes (warship, merchant), eight headings (dead ahead, dead astern, 45–, 90–, 135– port/starboard), two ranges (near, far), and three visibilities (clear, haze, fog) (Figure 2).

SAM advice was accurate on 50% of occasions (48 of 96), with equal numbers of errors made by ship class (*n* = 24) and heading (*n* = 24). To simulate real-world automation image recognition reliability (see Baker & Elder, 2022), SAM was 100% reliable in clear visibility, 50% reliable in haze, and 0% reliable in fog. SAM was also 67% reliable at near range and 33% reliable at far range. SAM only ever made errors by class or heading—never both at once (Table 1).

**Table 1.** SAM Performance by Ship Class (W = Warship, M = Merchant), Ship Factor (Class, Heading), Range (Near, Far), and Visibility (Clear, Haze, Fog).

SAM Performance	Factor	Visibility												*Total*
		Clear				Haze				Fog				
		Near		Far		Near		Far		Near		Far		
		W	M	W	M	W	M	W	M	W	M	W	M	
Accurate	Class and heading	8	8	8	8	8	8	—	—	—	—	—	—	48
Inaccurate	Class	—	—	—	—	—	—	4	4	4	4	4	4	24
	Heading	—	—	—	—	—	—	4	4	4	4	4	4	24
**Total**		8	8	8	8	8	8	8	8	8	8	8	8	96

Participants reviewed each ship image and received advice from SAM that could be accepted or rejected. If rejected, participants then needed to manually enter both ship class (two options) plus heading (eight options) using the SAM override panel (Figure 4).

#### Anthropomorphic Voice

Voice recordings of an adult male were used to create three voices (example audio at: https://www.youtube.com/watch?v=79o1ehXbtb8). The two ship classifications and eight ship headings were recorded three times—in monotone, with upward declarative inflection, and with downward interrogative inflection. To emulate a resonant robotic voice, recordings were mixed with duplicate tracks, one increased in pitch, without changing tempo, by half a semitone and time-shifted forwards by 50 ms, and one decreased by half semitone and time-shifted backwards by 50 ms. In the *monotone* condition, SAM voiced both ship class and heading information in monotone. In the *meaningless* condition, SAM randomly voiced ship class in either a downward declarative inflection or upward interrogative inflection, and then voiced heading in the opposite inflection. In the *meaningful* condition, SAM communicated confidence when accurate by voicing both ship class and heading with downward declarative inflections. When inaccurate, SAM communicated uncertainty by upward interrogative inflections for ship class or heading, depending on which factor was inaccurate, and a downward declarative inflection for the other factor that was accurate. In all three conditions, vocal SAM advice was reinforced by corresponding ship class icon and heading arrows appearing on screen directly below the image of SAM.

#### Anthropomorphic Appearance

**Figure 3.**
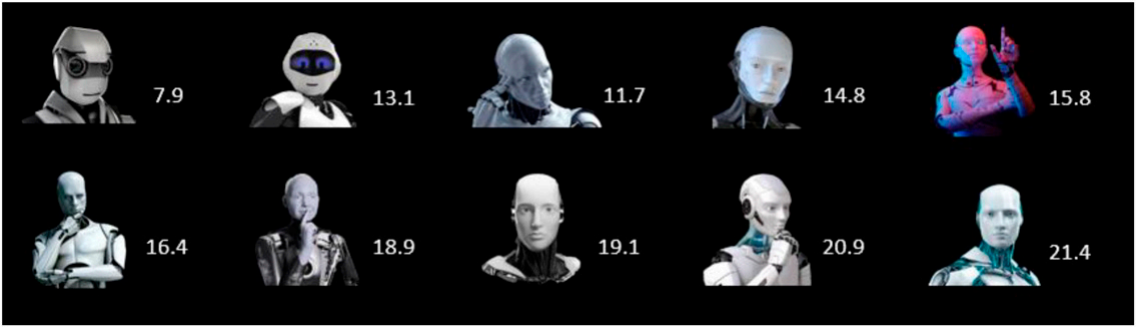
Rank-ordered comparison scores of ten robot faces (in 45 pairs) with *n* = 50 participants responding to the question ‘Which of these two robots looks more competent and trustworthy?’ The robot images are in order from least to most often selected with the avatar on the bottom-right adopted for the present experiment.

*Avatar* SAM was systematically selected from ten potential images to identify an image with social cues that appeared most competent and trustworthy. A sample of 50 *Amazon Mechanical Turk* workers were paid $0.50 each to participate in a four-minute survey. Participants viewed all possible pairs of the ten images (45 pairs) and were asked ‘which of these two robots looks more competent and trustworthy?’ This resulted in a mean rank-ordering of the ten images, with the most frequently selected image being adopted for the *avatar* SAM condition (Figure 3).

**Figure 4.**
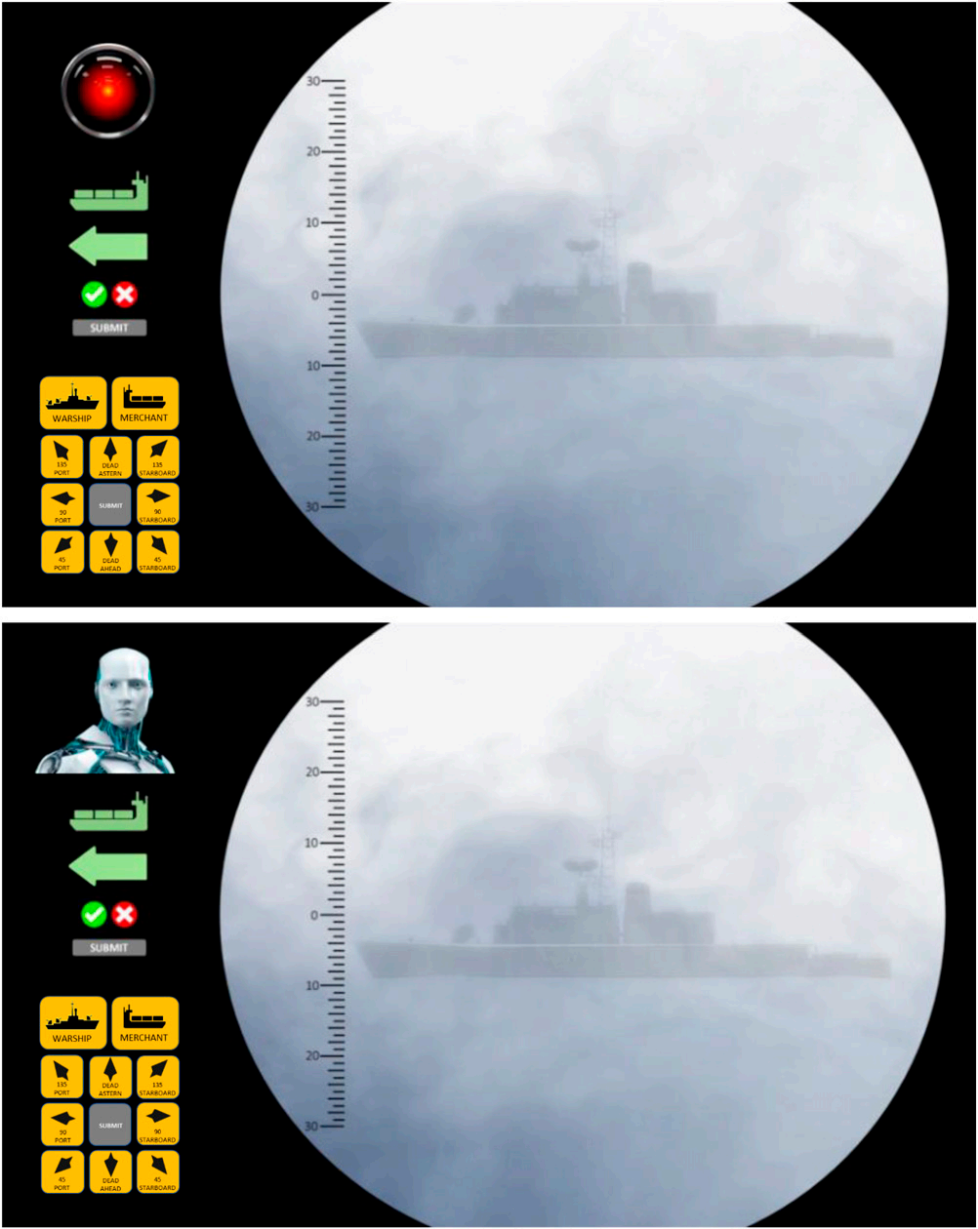
Anthropomorphic *appearance* conditions: *eye* (top) and *avatar* (bottom). The examples depict a warship at close range and in fog heading 90° port. Due to the fog, SAM predictably misclassifies the ship is a *merchant*. Participants could reject the advice by clicking the cross, causing the override buttons to appear, allowing the participant to manually enter *warship* heading 90-port.

For *Eye* SAM, a single camera lens was selected with negligible anthropomorphic properties—other than impassive regard—inspired by the antagonist HAL9000 from *2001: A Space Odyssey* (Figure 4).

### Measures

#### Subjective Trust

Subjective trust was assessed using the *Trust of Autonomous Systems Test* (TOAST), a psychometrically validated nine-item measure based upon a two-factor structure of system performance and system understanding (Wojton et al., 2020).

#### Trust Calibration

Trust calibration (accuracy of automation use) was a behavioural measure of d-prime (*d*'), representing the z-score of the proportion of SAM advice appropriately accepted by participants (hits) less the proportion accepted inappropriately (false alarms).

#### Confidence in Expectations

Participants were asked to subjectively rate their level of confidence in each instance of SAM advice that they accepted along a seven-point scale, to reflect their confidence in expectations when accepting SAM advice.

#### Anthropomorphism

The *Godspeed Anthropomorphism* measure (Bartneck et al., 2009) was incorporated to measure participant’s perception of SAM’s anthropomorphism. The measure features five 5-point scales that ask participants to rate along a continuum from fake–natural, machinelike–humanlike, unconscious–conscious, artificial–lifelike, and rigid–elegant. Participant ratings were then averaged to form an aggregated score of overall perceived anthropomorphism.

#### Attention Check

Two attention check items were inserted into the TOAST questionnaire in a randomised order that instructed participants ‘Please select *Generally Agree*’ and ‘Please select *Somewhat Disagree*’. Participants who did not follow these instructions were considered insincere respondents and their data were excluded.

### Procedure

Participants received 10 minutes’ training to familiarise themselves with the concepts of ship classification and heading, plus instructions on how to identify ship class and heading. Warships could be identified by gun turrets forward and a radar tower midship. Merchant vessels featured shipping containers and/or cranes forward, and lifeboats aft. Participants were then introduced to SAM and practiced accepting or rejecting SAM advice. Participants next practiced using the override interface to manually enter ship classifications/headings. Participants were only allowed to proceed to the actual experiment once they had demonstrated the ability to correctly enter class and heading information using the manual override panel for five consecutive ship images in clear conditions.

All participants were warned ‘SAM is not 100% accurate’ but the actual level of SAM reliability remained undisclosed. Participants were additionally advised ‘*SAM works well in clear conditions but struggles in hazy conditions, and super-struggles in fog, especially at far ranges. To this day, humans remain far better than computers at image recognition tasks in poor visual conditions*’. Participants randomly allocated to the *meaningful* inflection condition were also instructed that SAM expressed *certainty* and *uncertainty* and they were required to practice identifying three declarative upward inflections and three interrogative downward inflections, in a randomised order, until they demonstrated the ability to correctly differentiate between the two inflections by responding accordingly.

Upon completion of training, participants were advised they would classify 96 ships and receive immediate feedback after submitting each answer. To incentivise task performance, participants were advised that at the end of the experiment, they would receive a ranking of their performance against previous participants. Participants viewed the ships in a randomised order, either accepting SAM’s advice, or rejecting it and manually entering both ship class and heading. After viewing and responding to all the ship images, participants completed the TOAST and Godspeed questionnaires.

## Results

Participants’ responses were stored on the PsyToolkit server, downloaded, cleaned, and merged using RStudio (v.2023.06.1 + 524). In place of *p*-values, we report Bayes Factors (BFs), which can be interpreted as the strength of evidence for one hypothesis over another (e.g., a BF of 10 means one hypothesis is 10 times more likely than the other) (Kruschke & Liddell, 2018). To distinguish evidence for the alternate versus null hypotheses we report BF_10_ when evidence favours the alternate hypothesis, and BF_01_ when evidence favours the null hypothesis. We use the original labelling conventions of Jeffreys (1961, p. 432) where a BF of 1 ≤ 3 is considered ‘not worth more than bare mention’ [abbreviated hereafter to ‘negligible’], 3 ≤ 10 ‘substantial’, 10 ≤ 30 ‘strong’, 30 ≤ 100 ‘very strong’, and 100+ ‘decisive’.

The single outcome variable measures (Godspeed and TOAST) were analysed via two-way Bayesian ANOVA using jamovi (v.2.3.28) and the jsp package (v.1.2.0), treating participant identification as a random factor. Repeated measures (*d*', confidence ratings) were averaged per 16 trials, creating six time blocks over the 96 trials. Mean *d'* scores and confidence ratings per block were analysed with the gamjli package (v.2.6.6) using repeated-measures Bayes ANOVA, treating *appearance* and *inflection* as between-subjects variables, *block* as a within-subject variable, and participant identification as a cluster variable.

### Attention Check

Three participants failed both attention check items embedded within TOAST. An additional two participants spent more than 2 hours completing the online experiment, whereas the average completion time was 33.1 minutes (*SD* = 5.9). Data from these five participants was excluded, leaving a final sample of 93 participants.

### Manipulation Check

#### Anthropomorphism

**Figure 5.**
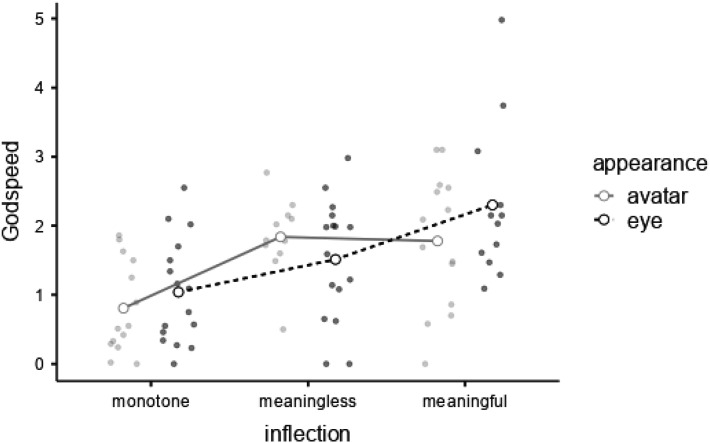
Mean scores on the *Godspeed Anthropomorphism* impression scale by *inflection* and *appearance* (with all observations).

A 2 (*appearance*) × 3 (*inflection*) Bayesian ANOVA examining mean *Godspeed Anthropomorphism* impression scale responses indicated substantial evidence that *eye* ( $\bar{X}$ = 1.560, *SD* = 1.020) and *avatar* ( $\bar{X}$ = 1.460, *SD* = .886) were rated the same, BF_M_ = 8.64 × 10^−4^, BF_01_ = 3.984. For *inflection*, there was decisive evidence for a main effect, BF_M_ = 8.879, BF_10_ = 800.820. Post hoc comparisons provided very strong evidence that *monotone* inflections ( $\bar{X}$ = .931, *SD* = .721) were rated less anthropomorphic than *meaningless* inflections ( $\bar{X}$ = 1.652, *SD* = .761), BF_10_ = 57.364, decisive evidence that monotone inflections were rated less anthropomorphic than *meaningful* inflections ( $\bar{X}$ = 2.020, *SD* = 1.060), BF_10_ = 617.136, and negligible evidence that *meaningless* and *meaningful* inflections were rated similarly, BF_01_ = 1.492. Examining models by inclusion Bayes factors suggested a model with no interaction between main effects was over twice as likely compared to an interaction between *appearance* and *inflection, BF*_*inclusion*_ = .498 (Figure 5).

### Trust

#### Subjective Trust (TOAST)

**Figure 6.**
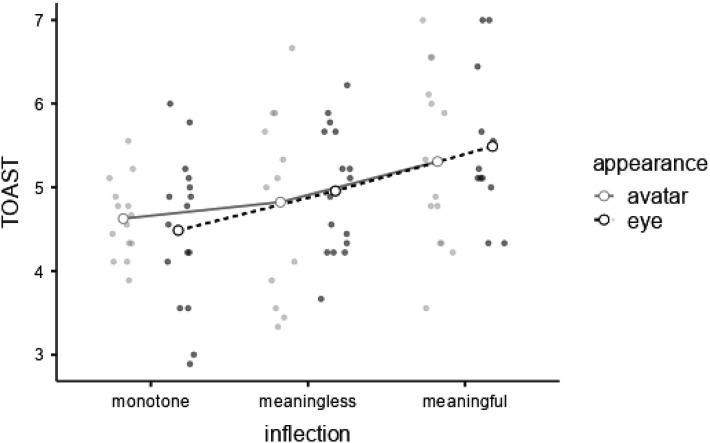
Mean scores on the *Trust of Automated Systems Test* (TOAST) by *inflection* and *appearance* (with all observations).

A 2 (*appearance*) × 3 (*inflection*) Bayesian ANOVA examining TOAST scores suggested substantial evidence against *eye* ( $\bar{X}$ = 4.93, *SD* = .923) and *avatar* ( $\bar{X}$ = 4.93, *SD* = .940) differing, BF_M_ = .043, BF_01_ = 4.389 (Figure 6). For *inflection*, there was strong evidence for a main effect, BF_M_ = 11.130, BF_10_ = 15.682. Post hoc comparisons provided negligible evidence that *meaningless* inflections ( $\bar{X}$ = 4.90, *SD* = .911) were rated similarly to *monotone* inflections ( $\bar{X}$ = 4.55, *SD* = .727), BF_01_ = 1.271, very strong evidence of *meaningful* inflections ( $\bar{X}$ = 5.39, *SD* = .969) being rated higher than *monotone* inflections, BF_10_ = 55.556, and negligible evidence that *meaningless* and *meaningful* inflections were rated differently, BF_10_ = 1.242. Examining models by inclusion Bayes factors suggested a model with no interaction between main effects was over 50-times more likely compared to an interaction between *appearance* and *inflection, BF*_*inclusion*_ = .019 (Figure 6).

#### Calibrated trust (*d'*)

A 2 (*appearance*) × 3 (*inflection*) × 6 (*time period*) repeated-measures Bayesian ANOVA was used to compare the *d*' of each experimental group. There was negligible evidence of a main effect by *block*, BF_M_ = 7.27 × 10^−7^, BF_10_ = 1.075, and substantial evidence of no difference between *eye* ( $\bar{X}$ = 2.87, *SD* = 1.57) and *avatar* ( $\bar{X}$ = 3.06, *SD* = 1.79), BF_M_ = 1.86 × 10^−7^, BF_01_ = 3.659. However, there was decisive evidence of a main effect by *inflection*, BF_M_ = 9.573, BF_10_ = 9.24 × 10^6^. Post hoc comparisons suggested there was decisive evidence of better *d'* for *meaningful* ( $\bar{X}$ = 4.54, *SD* = 1.33) compared to both *monotone* ( $\bar{X}$ = 2.06, *SD* = 1.17), BF_10_ = 1.20 × 10^23^, and *meaningless* ( $\bar{X}$ = 2.16, *SD* = 1.39), BF_10_ = 1.03 × 10^17^, and substantial evidence of no difference between the *monotone* and *meaningless* groups, BF_01_ = 4.651.

**Figure 7.**
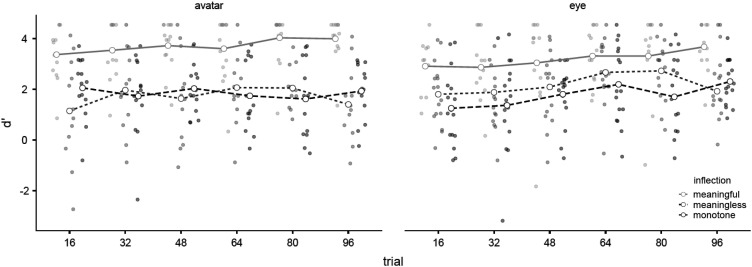
Mean *d*' by *block*, *appearance,* and *inflection* (with all observations).

Examination of inclusion Bayes factors suggested a model without any interactions between main effects is more likely than models with an interaction for *trial* × *inflection*, BF_inclusion_ = .108, *trial* × *appearance*, BF_inclusion_ = .013, or *trial* × *inflection* × *appearance*, BF_inclusion_ = 8.93 × 10^−4^. However, as one of the predictions of HATEM was that trust calibration would improve over time for *meaningful*, but not *meaningless* or *monotone* conditions, separate Bayes paired-sample t-tests were used to compare the *d*' of each condition for the first trial block (1–16) compared to last trial block (80–96). For *meaningful* participants there was strong evidence of improvement between the first block ( $\bar{X}$ = 3.16, *SD* = 1.01) compared to last block ( $\bar{X}$ = 3.85, *SD* = .91), BF_−0_ = 16.7. In contrast, there was substantial evidence for no improvement for *meaningless* participants between the first block ( $\bar{X}$ = 1.59, *SD* = 1.63) and last block ( $\bar{X}$ = 1.70, *SD* = 1.64), BF_0-_ = 3.42, and negligible evidence of an improvement for *monotone* participants between the first block ( $\bar{X}$ = 1.63, *SD* = 1.39) and last block ( $\bar{X}$ = 2.13, SD = .98), BF_−0_ = 1.08 (Figure 7).

#### Confidence in Expectations

**Figure 8.**
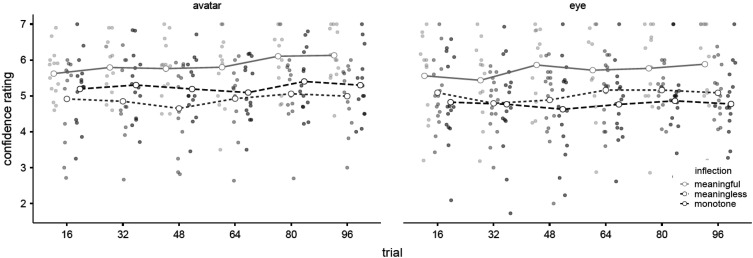
Mean *confidence ratings* of *SAM advice* by *appearance*, *inflection,* and *block* (with all observations).

A 2 (*appearance*) × 3 (*inflection*) × 6 (*block*) repeated-measures Bayesian ANOVA was used to compare the mean confidence rating of each experimental group. There was negligible evidence of a main effect by *block*, BF_M_ = .200, BF_10_ = 1.79, and negligible evidence of no difference by *appearance* between *eye* ( $\bar{X}$ *=* 5.17, *SD =* 1.10) and *avatar* ( $\bar{X}$ *=* 5.36, *SD =* .99), BF_M_ = .066, BF_01_ = 1.67. However, there was very strong evidence of a main effect by *inflection*, BF_M_ = 4.41, BF_10_ = 32.34. Post hoc comparisons suggested decisive evidence of greater confidence for *meaningful* ( $\bar{X}$ *=* 5.79, *SD =* .99) compared to *monotone* ( $\bar{X}$ *=* 4.99, *SD =* 1.05), BF_10_ = 1.31 × 10^9^, and *meaningless* ( $\bar{X}$ *=* 4.97, *SD =* .92), BF_10_ = 4.26 × 10^10^, and substantial evidence of no difference between the *monotone* and *meaningless* groups, BF_01_ = 8.32. Bayes paired-sample t-tests were used to compare the *confidence in expectations* of each condition for the first trial block (1–16) compared to last trial block (80–96). For *meaningful* participants there was substantial evidence of improvement between the first block ( $\bar{X}$ = 5.59, *SD* = .93) compared to last block ( $\bar{X}$ = 6.02, *SD* = .99), BF_-0_ = 7.46. In contrast, there was substantial evidence of no improvement for either *meaningless* participants between the first block ( $\bar{X}$ = 5.02, *SD* = .20) and last block ( $\bar{X}$ = 5.05, *SD* = 1.52), BF_0-_ = 4.14, or *monotone* participants between the first block ( $\bar{X}$ = 5.00, *SD* = 1.09) and last block ( $\bar{X}$ = 5.02, *SD* = .19), BF_-0_ = 4.67 (Figure 8).

#### Correlations

Consistent with the predictions of HATEM, participants with higher trust calibration (*d’*) had higher confidence ratings of SAM advice, *r* = .546, BF_+0_ = 3.99 × 10^5^, providing decisive evidence for convergent validity between these trust measures. Tellingly, the positive correlation was decisive for *meaningful* participants, *r* = .574, BF_+0_ = 41.61, very strong for *meaningless*, *r* = .515, BF_+0_ = 19.60, but negligible for *monotone*, *r* = .183, BF_+0_ = .587. Similarly, there was very strong overall evidence that trust calibration (*d’*) was positively correlated with subjective trust (TOAST), *r* = .360, BF_+0_ = 70.0. For *meaningful* participants, the correlation was very strong, *r* = .574, BF_+0_ = 41.61, but there was substantial evidence of no correlation for *meaningless*, *r* = −.001, BF_0+_ = 4.38, and negligible evidence for *monotone* participants, *r* = .221, BF_0+_ = 1.31. Finally, there was negligible evidence that subjective confidence ratings of SAM advice were positively correlated with TOAST, *r* = .204, BF_+0_ = 1.46. For *meaningful* participants, evidence of a positive correlation was negligible, *r* = .368, BF_+0_ = 2.37. In contrast, there was substantial evidence of no correlation for either *meaningless*, *r* = −.147, BF_01_ = 3.26, or *monotone* participants, *r* = .002, BF_01_ = 4.41.

## Discussion

The present study applied a new theoretical framework, the HATEM, to investigate the effect of anthropomorphism on HAT trust. Two manipulations of anthropomorphism were explored. The first was the most commonly-reported manipulation within the extant literature—superficial anthropomorphic manipulation by appearance. Ratings of perceived competence and trustworthiness were used to select an image for *Avatar SAM* that most likely conveyed social cues of trustworthiness and competence, while the image of *Eye SAM* was selected to be devoid of any human features that might convey social cues.

Unexpectedly, there was substantial evidence to suggest that for the *Godspeed* measure, participants’ anthropomorphic impressions of the *eye* and *avatar* conditions were the same, suggesting this manipulation was unsuccessful. However, this may plausibly be explained by the fact that previous studies reporting significant differences by appearance using *Godspeed* tended to use within-subject designs (e.g., de Visser et al., 2016, 2017, 2018) while studies using between-subject designs—like ours—have failed to detect a difference by appearance manipulation (e.g., Onnasch & Hildebrandt, 2022; Roesler et al., 2020).

To test this possibility, we conducted a follow-up, within-subjects, online survey of *n* = 17 participants (age $\bar{X}$ = 42.9, *SD* = 14.1, 41.2% male, 58.8% female) who completed the *Godspeed Anthropomorphism* questionnaire for both SAM *eye* and *avatar* images. A Bayesian paired-samples *t* test suggested decisive evidence of participants rating *avatar* ( $\bar{X}$ = 16.1, *SD* = 3.93) as more anthropomorphic than *eye* SAM ( $\bar{X}$ = 9.94, *SD* = 3.67)*,* BF_−0_ = 3499. This suggests the *Godspeed* measure is less discriminant for between-subject designs when participants lack comparison anchors—as previously observed in between-subject studies that also failed to detect differences for the *Godspeed* measure for appearance manipulation (Onnasch & Hildebrandt, 2022; Roesler et al., 2020).

The findings of Yee et al. (2007) led us to predict that the greater perceived anthropomorphism of *avatar* could engender assumptions of greater human-like competence resulting in increased subjective trust compared to *eye*, but we found no evidence of differences between *Avatar* and *Eye* SAM for subjective trust or confidence in expectations. Yee et al. (2007) concluded that behavioural trust measures are largely insensitive to manipulations by anthropomorphic appearance, and HATEM similarly predicted anthropomorphic appearance would have no discernible impact on behavioural trust. In line with this, we found no evidence of a difference in trust calibration between *avatar* and *eye* SAM. These outcomes replicate recent anthropomorphic appearance studies that also reported no differences on behavioural trust measures (e.g., de Visser et al., 2017; Kulms & Kopp, 2019).

The second manipulation compared trust measures by differences in anthropomorphic voice. On the basis of the Yee et al. (2007) meta-analysis, we predicted that *meaningful* and *meaningless* SAM could be subjectively trusted more than *monotone* SAM because they are more anthropomorphic in their resemblance to human-like inflections, but we only found evidence that *meaningful* SAM was rated subjectively more trustworthy than *monotone* SAM. HATEM further predicted that *meaningless* SAM, despite featuring an anthropomorphic voice, would hold no predictive advantage over *monotone* SAM and would therefore be no more objectively trusted. In line with this, we found no evidence of differences between *meaningless* and *monotone* conditions on the two objective trust measures (*d’* and confidence). HATEM also predicted that *meaningful* SAM, who naturalistically communicated useful information to help predict automation error, would result in enhanced objective trust compared to both *monoton*e and *meaningless* SAM. This prediction was supported decisively, with participants working with *meaningful* SAM evidencing better calibrated trust (*d’*) and confidence in SAM advice compared to their counterparts.

The lack of evidence for differences in trust measures between *monotone* and *meaningless* SAM were similar to a lack of evidence for differences between *avatar* and *eye* SAM. This reflects that none of these conditions provided helpful information to allow participants to predict SAM errors. In comparison, *meaningful* SAM featured the same anthropomorphic features as *meaningless* SAM but used these to better effect by naturalistically communicating useful information to participants, empowering them to better predict automation error. According to the *Godspeed* measure, there was decisive and very strong evidence respectively that participants considered *meaningful and meaningless* SAM more anthropomorphic than *monotone* SAM, while there was negligible evidence that *meaningless* SAM and *meaningful* SAM were rated differently. This is a useful result as it suggests participants viewed the inclusion of voiced declarative and interrogative inflections as more humanlike than *monotone* SAM—but also that participants viewed *meaningless* SAM as similarly anthropomorphic to *meaningful* SAM. As such, any differences in trust measures between the two inflected voice conditions can plausibly be attributed to the communicative value of the inflections, rather than their emulation of humanness per se. The fact that the *Godspeed* measure detected differences by *inflection* but not by *appearance* also suggests that when rating anthropomorphism participants were far more likely to attend to the voice component of SAM rather than his appearance.

There was also strong evidence supporting the HATEM prediction that trust calibration (*d’*) and confidence when accepting SAM advice would increase between the beginning and end of the experiment for individuals provided *meaningful* but not *monotone* or *meaningless* inflections. These outcomes support the assumption of HATEM that trust calibration and confidence will improve with task experience if humans have access to meaningful information that allows them to predict automation performance, and that task experience will make no difference to behavioural trust when meaningful information is absent. Finally, the prediction of HATEM that subjective and objective trust measures should be positively correlated was decisively supported, in line with the assumption of HATEM that trust is driven by the difference (closeness) between experienced automation reliability and expected automation reliability (prediction error). This prediction was underscored by very strong evidence of a positive correlation between subjective and objective trust measures when the information imparted by the anthropomorphic manipulation was meaningful, and negligible evidence of a correlation when it was not.

We expect that the key prediction of HATEM regarding anthropomorphism—that it will not improve trust calibration unless naturalistically communicating information to facilitate human prediction of automation failures—will be generalizable across different task domains, such as driving (Lee et al., 2023; Manchon et al., 2021), air traffic control (Bowden et al., 2021), and human-swarm interaction (Capiola et al., 2022). Further research is clearly required to test these assertions regarding generalizability, but at a minimum the current outcomes emphasize that attempting to manipulate human trust via the inclusion of superficial anthropomorphism may potentially have little effect on HAT trust.

It is also noteworthy that the relatively low automation reliability for the present experiment (50%) is well below the 70% threshold traditionally assumed necessary to achieve human-automation team trust (Lee & See, 2004; Wickens & Dixon, 2007). It is therefore telling that there was decisive evidence that participants in the *meaningful* group had greater HAT trust calibration, subjective trust, and confidence in accepted automation advice than their *meaningless* and *monotone* counterparts. This supports the theoretical underpinnings of HATEM that automation predictability is more important for calibrating HAT trust than automation reliability. That said, further research is required to test the potential theoretical boundary conditions of HATEM via manipulation of other factors known to affect HAT trust, such as reliability and transparency.

Enthusiasm for anthropomorphism remains seemingly irrepressible within the HAT literature despite recently published papers consistently failing to demonstrate benefits and some even finding negative effects (e.g., Christoforakos et al., 2021; Gray et al., 2022; Jensen, 2021; Onnasch & Hildebrandt, 2022). Shneiderman (2022) strongly advises against the use of human form and anthropomorphism in robots, in order to avoid overtrust, and warns humans should only ever consider automation as a tool rather than teammate. The findings of the present study somewhat contradict this contention; HATEM provides a clear and testable theoretical mechanism to identify poorly versus well-conceived anthropomorphism. The present study demonstrates that anthropomorphism retains potential as an efficient, naturalistic medium to communicate useful information from automation to humans, to enhance—rather than hinder—trust calibration. Crucially, this does not preclude humans from still viewing anthropomorphic automation as a tool but rather highlights the importance of human contextual understanding of automation teammate limitations.

Future investigators, and designers alike, who wish to incorporate anthropomorphism into automation, should be very clear from the outset what specific and useful information they wish the anthropomorphism to convey to the human operator. Even more specifically, they should consider how such information can be used by human teammates to improve their predictive understanding of automation failure.

## Key Points

- Evidence that anthropomorphism improves behavioural trust remains equivocal.
- Our results provide evidence that superficial anthropomorphism—such as humanlike appearance and meaninglessly inflected voices—does not necessarily increase subjective trust or behavioural trust.
- A naturalistic communication device to convey automation certainty and uncertainty—via voice inflections—demonstrably increased subjective trust and behavioural trust, regardless of level of anthropomorphism appearance.
- Designers interested in incorporating anthropomorphic features into automation need to consider what contextually useful information such features will communicate to the human operator that will allow them to accurately predict automation performance.

## ORCID iDs

Owen B. J. Carter https://orcid.org/0000-0003-3223-1455

Shayne Loft https://orcid.org/0000-0002-5434-0348

Troy A. W. Visser https://orcid.org/0000-0003-3960-2263
